# Integrating multiparametric MRI radiomics and clinical models to assess sensitivity to neoadjuvant chemotherapy in breast cancer: A multicenter study

**DOI:** 10.1002/acm2.70347

**Published:** 2025-11-14

**Authors:** Xinyi Zeng, Jinxin Chen, Xianjun Zeng, Xiaofei Tang, Jidong Peng

**Affiliations:** ^1^ Ganzhou Institute of Medical Imaging Ganzhou Key Laboratory of Medical Imaging and Artificial Intelligence Medical Imaging Center Ganzhou People's Hospital The Affiliated Ganzhou Hospital of Nanchang University Ganzhou Hospital‐Nanfang Hospital Southern Medical University Ganzhou China; ^2^ The First Affiliated Hospital of Nanchang University Nanchang University Nanchang China; ^3^ Ganzhou Cancer Hospital Ganzhou China

**Keywords:** breast cancer, multiparametric MRI, neoadjuvant chemotherapy, radiomics, SHAP

## Abstract

**Objective:**

To develop and externally validate an interpretable multiparametric MRI‐based radiomic‐clinical model using Shapley Additive Explanations (SHAP) methodology for early prediction of breast cancer sensitivity to neoadjuvant chemotherapy (NAC).

**Methods:**

This retrospective multicentric study enrolled 223 breast cancer patients from three medical centers. Patients underwent pretreatment multiparametric MRI (DCE‐MRI and DWI sequences) with Miller‐Payne grades 4‐5 defining NAC‐sensitive. Manual tumor segmentation generated regions of interest for extracting 2,396 radiomic features per patient. Feature selection integrated reproducibility analysis (ICC > 0.7), univariable significance testing (*p* < 0.01), LASSO regression, and hierarchical clustering. A support vector machine (SVM) model incorporated optimized radiomic signatures and clinical variables. SHAP methodology provided global feature importance interpretation and individualized prediction explanations.

**Results:**

The integrated radiomic‐clinical model demonstrated superior performance to standalone clinical (AUC 0.720) and radiomic (AUC 0.833) models in the internal validation set, achieving an AUC of 0.904 (95% CI: 0.816–0.991). This advantage persisted in external validation (AUC 0.928, 95% CI: 0.874–0.982). SHAP analysis identified wavelet_HHL_glcm_Correlation_DCE as the predominant predictive feature, with high values significantly correlating to NAC‐sensitive. A clinical nomogram translated model outputs into quantifiable risk probabilities, where total scores ≥130 indicated > 95% sensitivity likelihood.

**Conclusion:**

The SHAP‐explainable radiomic‐clinical model provides a clinically applicable, noninvasive tool for pretreatment stratification of NAC sensitivity. This approach enhances personalized therapeutic decision‐making while minimizing unnecessary treatment toxicity.

## 1 INTRODUCTION

Breast cancer ranks as the most commonly diagnosed malignancy and the second leading cause of cancer‐related death among women worldwide.^1^ Effective prevention, early diagnosis, and timely therapeutic intervention are crucial for halting cancer progression.^2^ Neoadjuvant chemotherapy (NAC) has become increasingly integrated with conventional surgery, often complemented by adjuvant therapies. Currently, NAC is widely recognized as a primary treatment strategy for patients with early‐stage, high‐risk, or locally advanced breast cancer.^3^ However, NAC is not universally beneficial. Approximately 30% of patients may exhibit poor or no response to treatment, while others can experience significant adverse effects, including systemic toxicity, liver and kidney impairment, and cardiac dysfunction.^4^ Consequently, early identification of NAC sensitivity in breast cancer is essential to optimize treatment pathways and explore alternative treatment options. Presently, the definitive assessment of tumor response relies on the histopathological examination of surgically resected specimens. The non‐invasive prediction of therapeutic efficacy and patient outcomes at an early stage remains a significant clinical challenge.

Radiomics, by extracting and analyzing numerous quantitative imaging features to characterize tumor heterogeneity, offers a promising approach to enhance our understanding of cancer pathophysiology and support clinical decision‐making.^5^ Emerging research demonstrates the considerable potential of radiomics for breast cancer diagnosis, molecular subtyping, prediction of NAC response, and prognostication.^6^, ^7^, ^8^ However, the clinical adoption of radiomic models is often hindered by concerns regarding their “black box” nature—specifically, the unclear internal mechanisms and lack of interpretability of the predictive features.^9^ This limited explainability significantly impedes the widespread clinical implementation of radiomics. The Shapley Additive Explanations (SHAP) method offers a potential solution to this barrier. SHAP provides a unified, game‐theory‐based framework for interpreting machine learning model predictions by quantifying the contribution of each feature to the output, defining a class of additive feature importance measures with strong theoretical foundations.^10^ Its key advantages include the ability to illustrate global feature importance, reveal the directional impact of features on model predictions, and explain individual predictions, thereby enhancing model transparency.^11^ ^,^ ^12^ Combining SHAP with radiomics holds promise for developing highly performing yet interpretable predictive models.^13^

Therefore, this study aims to develop and externally validate a multiparametric MRI (mpMRI)‐based model integrating radiomic features and clinical characteristics to predict NAC treatment sensitivity in breast cancer patients. Furthermore, we integrate the SHAP methodology to provide a comprehensive explanation and intuitive visualization of the model's predictive mechanisms. We hypothesize that the SHAP‐explainable radiomic‐clinical model will provide superior predictive accuracy with individualized, explainable predictions to aid clinical decision‐making for NAC treatment response.

## 2 MATERIALS AND METHODS

### 2.1 Patients

**FIGURE 1:**
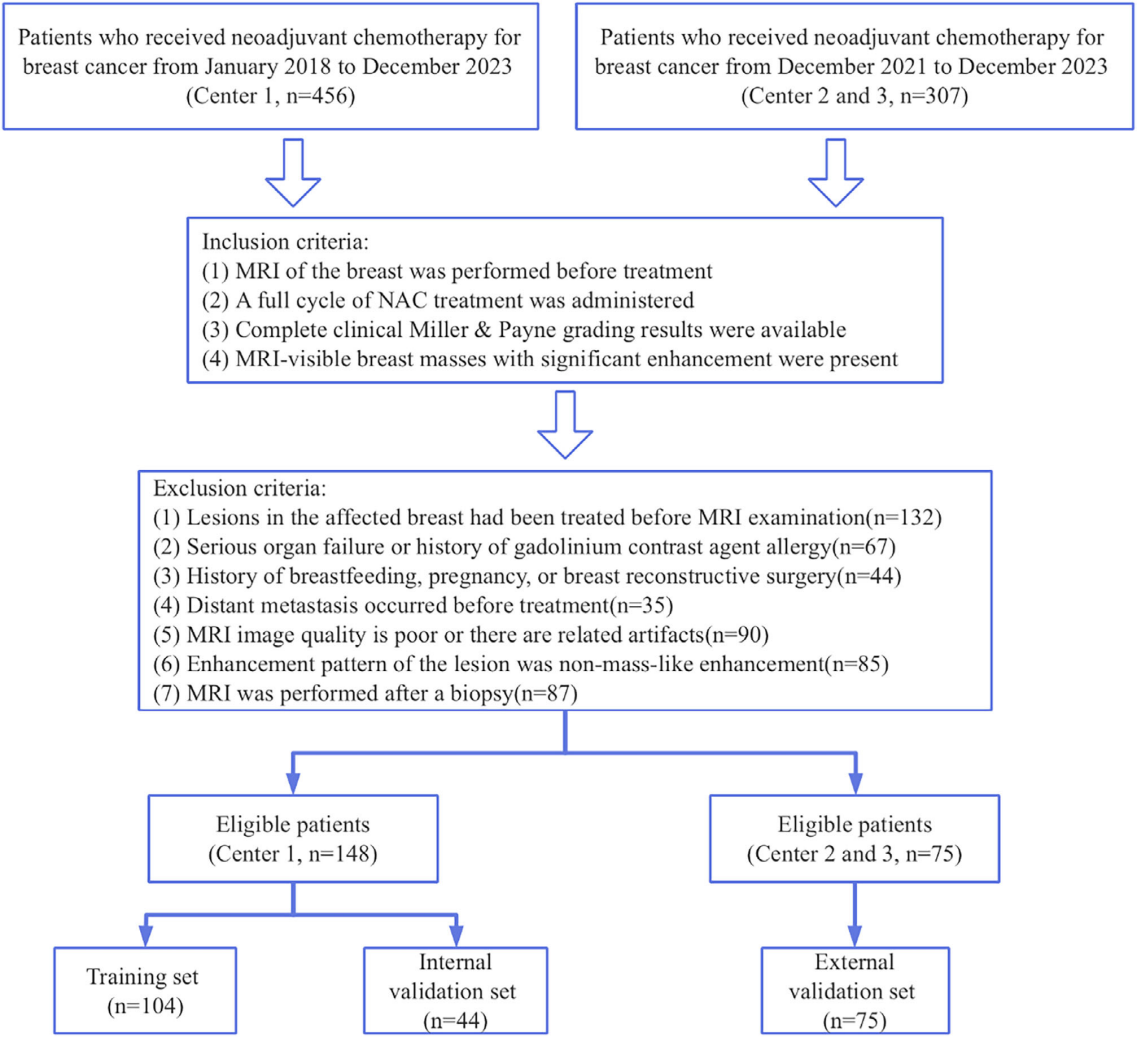
Flow chart of patient enrollment and grouping.

This retrospective clinical study was conducted at three medical centers. The study complied with the Declaration of Helsinki and obtained ethical approval from all participating institutions. A total of 223 breast cancer patients undergoing NAC were enrolled across the three centers. Inclusion criteria comprised: pretreatment breast MRI; completion of a full NAC cycle; availability of definitive clinical Miller‐Payne grading results; and presence of MRI‐visible breast masses exhibiting significant enhancement. Exclusion criteria included: prior treatment of affected breast lesions before MRI examination; severe organ failure or history of gadolinium‐based contrast allergy; breastfeeding, pregnancy, or breast reconstructive surgery history; distant metastasis pretreatment; MRI image quality is poor or there are related artifacts; non‐mass‐like enhancement patterns; or post‐biopsy MRI acquisition. Center 1 served as the training and internal validation set, while Center 2 and 3 constituted external validation set for model assessment (Figure 1).

### 2.2 Selection of clinical features

Baseline information and relevant clinical indicators were collected for all included cases, encompassing age, pathological Miller‐Payne grade, estrogen receptor (ER), progesterone receptor (PR), human epidermal growth factor receptor 2 (HER‐2), Ki‐67 index, pathological type, molecular subtype, lesion location, lesion multiplicity, lesion margins, lesion morphology, long diameter, short diameter, glandular tissue type, time‐signal intensity curve (TIC) pattern, breast parenchymal enhancement characteristics, axillary lymph node metastasis status, skin thickening, and subcutaneous fat layer opacity. The listed biomarkers were determined from pretreatment tumor specimens using immunohistochemistry (IHC) and/or fluorescence in situ hybridization (FISH). ER and PR positive were defined as > 1% stained tumor cells. HER‐2 positive was defined as IHC 3+ or IHC 2+ with confirmed HER‐2 gene amplification by FISH.^14^ Ki‐67 expression was dichotomized using the St. Gallen 2015 Consensus threshold (≥20% = high expression; < 20% = low expression).^15^ Luminal A subtype is characterized by ER positive, PR positive (≥20%), HER‐2 negative, and a Ki67 index of less than 20%. In contrast, the Luminal B subtype includes ER‐positive and/or PR‐positive (< 20%) tumors that are HER‐2 negative, as well as tumors that are positive for ER, PR, and HER‐2, or those with ER and PR positivity but a Ki67 level exceeding 20%. HER‐2 positive was defined as ER/PR negative and HER‐2 positive. The triple‐negative group was defined as ER/PR/HER‐2 negative.

### 2.3 NAC treatment protocol and efficacy evaluation

All patients received taxane and/or anthracycline‐based chemotherapy regimens according to NCCN guidelines; HER‐2‐positive patients additionally received anti‐HER‐2 targeted therapy. Pathological response to NAC was evaluated on postoperative specimens using the Miller‐Payne (MP) grading system:^16^ Grade 1, no reduction in infiltrating carcinoma cells; Grade 2, ≤ 30% reduction; Grade 3, 30%–90% reduction; Grade 4, > 90% reduction with minimal residual microscopic foci; Grade 5, no residual invasive carcinoma (ductal carcinoma in situ may remain). Patients with MP Grades 4‐5 were classified as NAC‐sensitive group, while MP Grades 1‐3 were defined as NAC‐insensitive group.^17^

### 2.4 MRI image acquisition, tumor segmentation, and feature extraction

**FIGURE 2:**
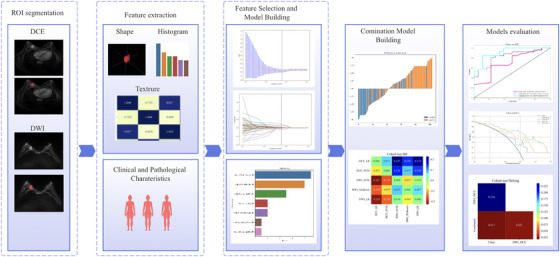
The workflow of the radiomics analysis.

Images were retrieved from Picture Archiving and Communication Systems (PACS). All patients underwent a standardized MRI protocol including axial dynamic contrast‐enhanced fat‐suppressed T1‐weighted imaging (DCE‐MRI) and axial diffusion‐weighted imaging (DWI) sequences (*b*‐value = 1000 s/mm^2^). DICOM‐format images were analyzed radiomically. A radiologist (2 years’ experience) manually annotated lesion boundaries slice‐by‐slice on DCE‐MRI and DWI using ITK‐SNAP software (http://www.itksnap.org), generating regions of interest (ROIs). Annotations were verified independently by two senior radiologists (10 and 15 years’ experience), with disagreements resolved by consensus. All patient identifiers and clinical data were anonymized during segmentation. Figure 2 illustrates the radiomics workflow. ROIs were processed using PyRadiomics (v3.7.12) to extract 1198 features per sequence, categorized into first‐order statistics, shape‐based, texture, and higher‐order features.

### 2.5 Feature selection and model building

Features demonstrating poor reproducibility (intraclass correlation coefficient [ICC] < 0.7) were excluded. Univariable analysis (Mann‐Whitney U‐test, *p* < 0.01) identified features with significant differences between NAC‐sensitive and insensitive groups. Multicolinear features (Spearman's correlation coefficient ≥|0.9|) were reduced by retaining the feature with superior diagnostic performance. Hierarchical clustering addressed redundancy (features with pairwise correlation > 0.95 within a cluster were merged, retaining the feature with maximal dynamic range). Least Absolute Shrinkage and Selection Operator (LASSO) logistic regression refined the optimal feature subsets for each model (clinical, radiomic, and radiomic‐clinical model) based on the training cohort. The optimal regularization parameter (λ) for the LASSO model was selected through 10‐fold cross‐validation based on the criterion of minimizing the mean squared error (MSE). As shown in Figure S1‐S2, the value of λ that yielded the minimum cross‐validated MSE was chosen (*λ* = 0.0596), ensuring a balance between model complexity and predictive performance. Support vector machine (SVM) models were subsequently constructed and evaluated. We used the RBF kernel for the SVM classifier. The hyperparameters were set as follows: the regularization parameter C was 1.0 and gamma was set to ‘scale’. The value for gamma = 'scale' is automatically calculated as 1 / (n_features * X.var()), where n_features is the number of features and X.var() is the variance of the training data.

### 2.6 Model explanation and visualization

To enhance model interpretability, significant clinical features and radiomic signatures were incorporated into a nomogram for visual predictive probability assessment. Additionally, SHAP analysis was employed to mitigate machine learning's “black‐box” effect. SHAP values, derived from coalitional game theory, quantify each feature's average marginal contribution to the model output across all potential combinations, enabling feature prioritization, and interpretability.^10^, ^11^, ^12^ Kernel SHAP explainers were implemented, treating patient features as “players” contributing to the prediction “payout”.^10^ ^,^ ^12^

### 2.7 Statistical analysis

All statistical analyses were performed using Python 4.1 and SPSS 25.0. Continuous variables, expressed as mean ± standard deviation (SD) were compared using unpaired two‐tailed t‐tests (normal distribution) or Mann‐Whitney U‐tests (non‐normal distribution). Categorical variables were analyzed using χ^2^ or Fisher's exact tests. Model performance was assessed using receiver operating characteristic (ROC) curves, specificity, sensitivity, precision, and area under the curve (AUC) with 95% confidence intervals (95% CI). Delong's test compared ROC curves between cohorts. Calibration curves evaluated model calibration accuracy. A two‐tailed p < 0.05 was considered statistically significant.

## 3 RESULTS

### 3.1 Clinical characteristics

As shown in Table 1, 148 eligible patients were enrolled in Center 1, which was divided into a training set (*n* = 104) and an internal validation set (*n* = 44) in a 7:3 ratio. A total of 75 eligible patients from Center 2 and 3 comprised the external validation set. Within the training set, statistically significant differences were observed between the sensitive and insensitive groups regarding ER status, PR status, HER‐2 status, molecular subtypes, nipple retraction, skin thickening, and subcutaneous fat. A clinical model was developed based on these significant univariate factors. This model yielded an AUC of 0.787 (95% CI: 0.702‐0.873) in the training set, 0.720(95% CI: 0.562‐0.879) in the internal validation set, and 0.740(95% CI: 0.627‐0.853) in the external validation set.

**TABLE 1:** Comparison of clinical‐pathological and MRI imaging features.

Features	Training set (*n* = 104)			Internal validation set (*n* = 44)			External validation set (*n* = 75)		
	Insensitive group (*n* = 46)	Sensitive group (*n* = 58)	*p* value	Insensitive group (*n* = 18)	Sensitive group (*n* = 26)	*p* value	Insensitive group (*n* = 37)	Sensitive group (*n* = 38)	*p* value
Age	50.11 ± 9.04	48.88 ± 7.93	0.462^c)^	50.28 ± 9.09	46.96 ± 8.17	0.213^c)^	50.73 ± 10.56	51.29 ± 10.15	0.816^c)^
ER			0.016^b)^			0.019^b)^			0.028^b)^
Negative	13(28.3%)	30(51.7%)		6(33.3%)	18(69.2%)		13(35.1%)	24(63.2%)	
Positive	33(71.7%)	28(48.3%)		12(66.7%)	8(30.8%)		24(64.9%)	14(36.8%)	
PR			0.002^b)^			0.005^b)^			0.014^b)^
Negative	18(39.1%)	40(69.0%)		7(38.9%)	21(80.8%)		17(45.9%)	29(76.3%)	
Positive	28(60.9%)	18(31.0%)		11(61.1%)	5(19.2%)		20(54.1%)	9(23.7%)	
Her2			0.018^b)^			0.005^b)^			<0.001^b)^
Low proliferation	24(52.12%)	17(29.3%)		14(77.8%)	9(34.6%)		30(81.1%)	12(31.6%)	
High proliferation	22(47.8%)	41(70.7%)		4(22.2%)	17(65.4%)		7(18.9%)	26(68.4%)	
Ki‐67			0.156^b)^			0.357^b)^			0.016^b)^
< 20%	6(13.0%)	3(5.2%)		3(16.7%)	1(3.8%)		7(18.9%)	0(0%)	
≥20%	40(87.0%)	55(94.8%)		15(83.3%)	25(96.2%)		30(81.1%)	38(100%)	
P63			0.837^b)^			0.376^b)^			0.695^b)^
Negative	39(84.8%)	50(86.2%)		18(100%)	23(88.5%)		35(94.6%)	34(89.5%)	
Positive	7(15.2%)	8(13.8%)		0(0%)	3(11.5%)		2(5.4%)	4(10.5%)	
P120			1.000^b)^			1.000^b)^			1.000^b)^
Negative	0(0%)	1(1.7%)		0(0%)	0(0%)		0(0%)	0(0%)	
Positive	46(100%)	57(98.3%)		18(100%)	26(100%)		37(100%)	38(100%)	
E‐cad			1.000^b)^			0.640^b)^			1.000^b)^
Negative	0(0%)	0(0%)		0(0%)	2(7.7%)		0(0%)	0(0%)	
Positive	46(100%)	58(100%)		18(100%)	24(92.3%)		37(100%)	38(100%)	
Location			0.563^b)^			0.329^b)^			0.612^b)^
Outer upper	18(39.1%)	25(43.1%)		8(44.4%)	11(42.3%)		18(48.6%)	17(44.7%)	
Outer lower	5(10.9%)	11(19.0%)		1(5.6)%	4(15.4%)		3(8.1%)	6(15.8%)	
Lower inner	2(4.3%)	3(5.2%)		2(11.1%)	5(19.2%)		0(0%)	2(5.3%)	
Upper inner	6(13.0%)	9(15.5%)		1(5.6%)	2(7.7%)		9(24.3%)	8(21.1%)	
Central area	10(21.7%)	7(12.1%)		3(16.7%)	4(15.4%)		5(13.5%)	3(7.9%)	
Whole breast	5(10.9%)	3(5.2%)		3(16.7%)	0(0%)				
FGT			0.219^b)^			0.369^b)^			0.417^b)^
Low FGT	40(87.0%)	45(77.6%)		16(88.9%)	19(73.1%)		22(59.5%)	27(71.1%)	
High FGT	6(13.0%)	13(22.4%)		2(11.1%)	7(26.9 %)		15(40.5%)	11(28.9%)	
Single or multiple lessions			0.418^b)^			1.000^b)^			1.000^b)^
Single	17(37.0%)	26(44.8%)		13(72.2%)	20(77.0 %)		25(67.6%)	26(68.4)	
Multiple	29(63.0%)	32(55.2%)		5(27.8%)	6(23.0%)		12(32.4%)	12(31.6%)	
Long diameter	40.83 ± 16.72	35.84 ± 15.15	0.190^a)^	39.83 ± 15.98	36.50 ± 18.21	0.256^a)^	42.30 ± 19.69	35.26 ± 15.76	0.092^a)^
Short diameter	30.98 ± 12.56	26.59 ± 10.88	0.157^a)^	25.39 ± 8.05	24.69 ± 10.24	0.357^a)^	29.35 ± 15.09	23.53 ± 7.89	0.060^a)^
Shape of the lump			0.421^b)^			1.000^b)^			1.000^b)^
Regular	11(23.9%)	18(31.0%)		7(38.9%)	10(38.5%)		10(27.1%)	10(26.3%)	
Irregular	35(76.1%)	40(69.0%)		11(61.1%)	16(61.5%)		27(72.9%)	28(73.7%)	
Boundary			0.298^b)^			0.135^b)^			0.097^b)^
Clear	2(4.3%)	7(12.1%)		0(0%)	5(19.2%)		13(35.1%)	6(15.8%)	
Vague	44(95.7%)	51(87.9%)		18(100%)	21(80.8%)		24(64.9%)	32(84.2%)	
Molecular subtype			0.069^b)^			0.002^b)^			<0.009^b)^
Luminal A	2(4.3%)	0(0%)		2(11.1%)	0(0%)		6(16.2%)	0(0%)	
Luminal B	31(67.5%)	29(50.0%)		10(55.6%)	8(30.8%)		19(51.4%)	15(39.5%)	
HER2+	7(15.2%)	18(31.0%)		0(0%)	13(50.0%)		5(13.5%)	15(39.5%)	
Triple‐negative	6(13.0%)	11(19.0%)		6(33.3%)	5(19.2%)		7(18.9%)	8(21.0%)	
Background Enhancement			1.000^b)^			0.303^b)^			0.320^b)^
Slight Enhancement	2(4.3%)	2(3.4%)		6(33.3%)	4(15.4%)		8(21.6%)	4(10.5%)	
Significant Enhancement	44(95.7%)	56(96.6%)		12(66.7%)	22(84.6%)		29(78.4%)	34(89.5%)	
Histologic subtype			0.838^b)^			1.000^b)^			0.198^b)^
Ductal carcinoma	44(95.6%)	57(98.3%)		18(100%)	25(96.2%)		34(91.9%)	33(86.8%)	
Lobular carcinoma	2(4.4%)	1(1.7%)		0(0%)	0(0%)		1(2.7%)	5(13.2%)	
Others	0(0%)	0(0 %)		0(0%)	1(3.8%)		2(5.4%)	0(0%)	
TIC			0.984^b)^			0.831^b)^			0.081^b)^
Inflow	1(2.2%)	1(1.7%)		0(0%)	0(0%)		0(0%)	0(0%)	
Platform	10(21.7%)	13(22.4%)		5(27.8%)	8(30.8%)		24(64.9%)	16(42.1%)	
Outflow	35(76.1%)	44(75.9%)		13(72.2%)	18(69.2%)		13(35.1%)	22(57.9%)	
Subcutaneous fat			0.002^b)^			0.063^b)^			0.848^b)^
Clear	13(28.3%)	34(58.6%)		4(22.2%)	13(50.0%)		9(24.3%)	11(28.9%)	
Muddy	33(71.7%)	24(41.4%)		14(77.8%)	13(50.0%)		28(75.7%)	27(71.1%)	
Thicken skin			0.002^b)^			0.329^b)^			0.417^b)^
Absent	15(32.6%)	37(63.8%)		7(38.9%)	14(53.8%)		22(59.5%)	27(71.1%)	
Present	31(67.4%)	21(36.2%)		11(61.1%)	12(46.2%)		15(40.5%)	11(28.9%)	
Nipple retraction			0.012^b)^			0.071^b)^			0.050^b)^
Absent	30(65.2%)	50(86.2%)		10(55.6%)	21(80.8%)		24(64.9%)	33(86.9%)	
Present	24(34.8%)	47(13.8%)		8(44.4%)	5(19.2%)		13(35.1%)	5(13.1%)	
Enlargement of axillary lymph nodes			0.490^b)^			0.347^b)^			0.203^b)^
Absent	5(10.9%)	9(15.5%)		4(22.2%)	13(50.0%)		9(24.3%)	4(10.5%)	
Present	41(89.1%)	49(84.5%)		14(77.8%)	13(50.0%)		28(75.7%)	34(89.5%)	

*Note*: ^a)^Independent sample non‐parametric test;

^b)^ Chi‐square test;

^c)^ Independent sample t‐test.

### 3.2 Radiomic feature selection

Figure S2 illustrates the LASSO coefficient profiles and feature selection path leading to the optimal features derived from tumor region imaging characteristics. Initially, 1198 features were extracted from DCE sequences and 1198 from DWI sequences, resulting in a combined pool of 2396 features. After feature selection, 8 features were retained for model development (Figure S3): five from DCE images (wavelet_HHL_glcm_ClusterShade, wavelet_HHL_glcm_Correlation, wavelet_LLH_firstorder_Skewness, wavelet_LLH_glcm_ClusterShade, log_sigma_5_0_mm_3D_glszm_SmallAreaLowGrayLevelEmphasis) and three from DWI images (wavelet_HLL_firstorder_Median, wavelet_HLL_glcm_Idn, wavelet_HLL_glszm_ZoneVariance). These features constituted the radiomics signature.

### 3.3 Performance of radiomic‐based models to assess NAC sensitivity

**FIGURE 3:**
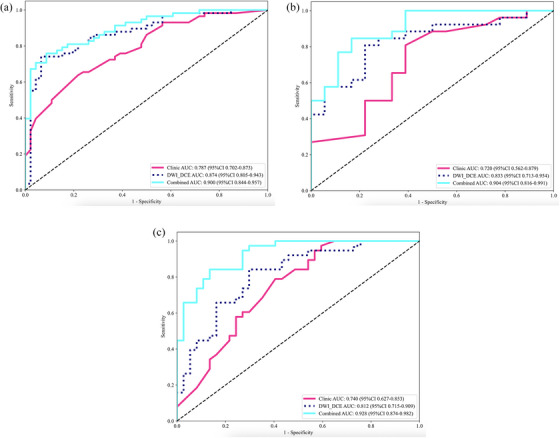
Results of clinical, radiomics and radiomics‐clinical ROC analysis in the training set (A), internal validation set (B), external validation set (C).

In the training set (Figure 3A, Table 2), the combined radiomics‐clinical model achieved an AUC of 0.900 (95% CI: 0.844–0.957), outperforming both the standalone clinical model (AUC 0.787, 95% CI: 0.702–0.873) and the standalone radiomics model (AUC 0.874, 95% CI: 0.806–0.943). Within the internal validation set (Figure 3B), the combined model maintained superior performance with an AUC of 0.904 (95% CI: 0.816–0.991), higher than the clinical model (AUC 0.720, 95% CI: 0.562–0.879), and the radiomics model (AUC 0.833, 95% CI: 0.713–0.954). In the external validation set (Figure 3B), the combined model's AUC was 0.928 (95% CI: 0.874–0.982), exceeding both the clinical model (AUC 0.740, 95% CI: 0.627–0.853) and the radiomics model (AUC 0.812, 95% CI: 0.715–0.909). Clinical decision curve (Figure S4) and calibration curve (Figure S5) showed that the radiomics‐clinical model generally provided greater clinical utility than either the clinical model or the integrated radiomics model. The *p* values of Delong test were both smaller than 0.05 when comparing the radiomic‐based models with the clinical model (Figure S6).

**TABLE 2:** The diagnostic efficacy of various models in predicting the sensitivity of breast cancer to neoadjuvant chemotherapy.

Cohort	Models	AUC(95%CI)	Sensitivity	Specificity	Accuracy	Precision
Training cohort	Clinical	0.787(0.702‐0.873)	0.638	0.783	0.702	0.787
	DCE_DWI	0.874(0.806‐0.943)	0.741	0.935	0.827	0.935
	Combined	0.900(0.844‐0.957)	0.759	0.913	0.827	0.917
Internal validation set	Clinical	0.720(0.562‐0.879)	0.808	0.611	0.727	0.750
	DCE_DWI	0.833(0.713‐0.954)	0.808	0.778	0.795	0.840
	Combined	0.904(0.816‐0.991)	0.846	0.833	0.841	0.880
External validation set	Clinical	0.740(0.627‐0.853)	0.789	0.595	0.693	0.667
	DCE_DWI	0.812(0.715‐0.909)	0.842	0.703	0.773	0.744
	Combined	0.928(0.874‐0.982)	0.842	0.865	0.853	0.865

Abbreviation: AUC: area under the curve; CI: confidence intervals.

### 3.4 Explanation and visualization of radiomic‐clinical model

**FIGURE 4:**
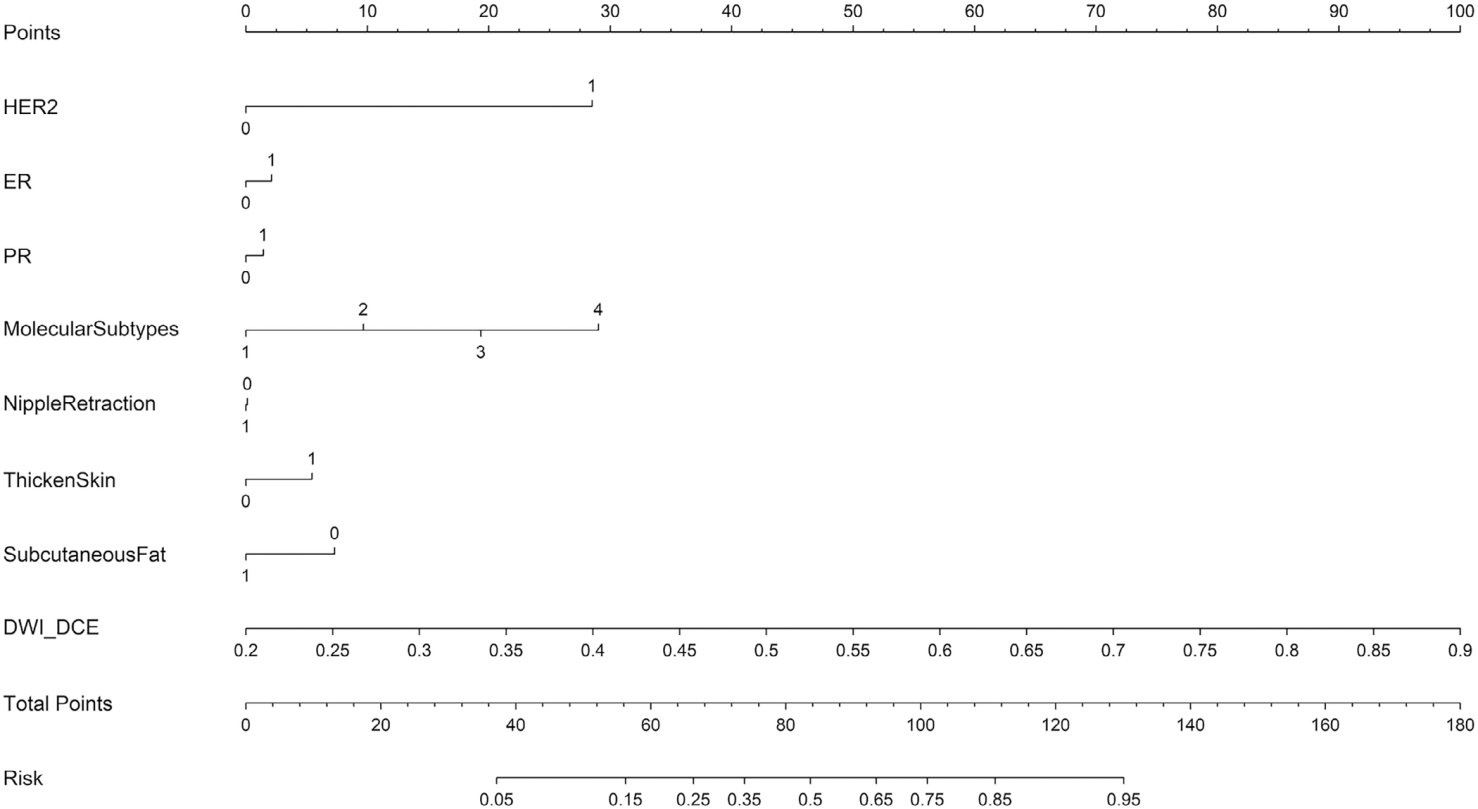
Nomogram of the radiomics‐clinical model.

A nomogram was constructed to quantify breast cancer risk through multi‐indicator integration (Figure 4). DWI_DCE contributed the largest weight to the total score, followed by molecular subtypes. A total nomogram score ≥130 (corresponding to a predicted risk probability ≥0.95) effectively identified high‐risk patients, offering a visual tool for personalized intervention decisions.

**FIGURE 5:**
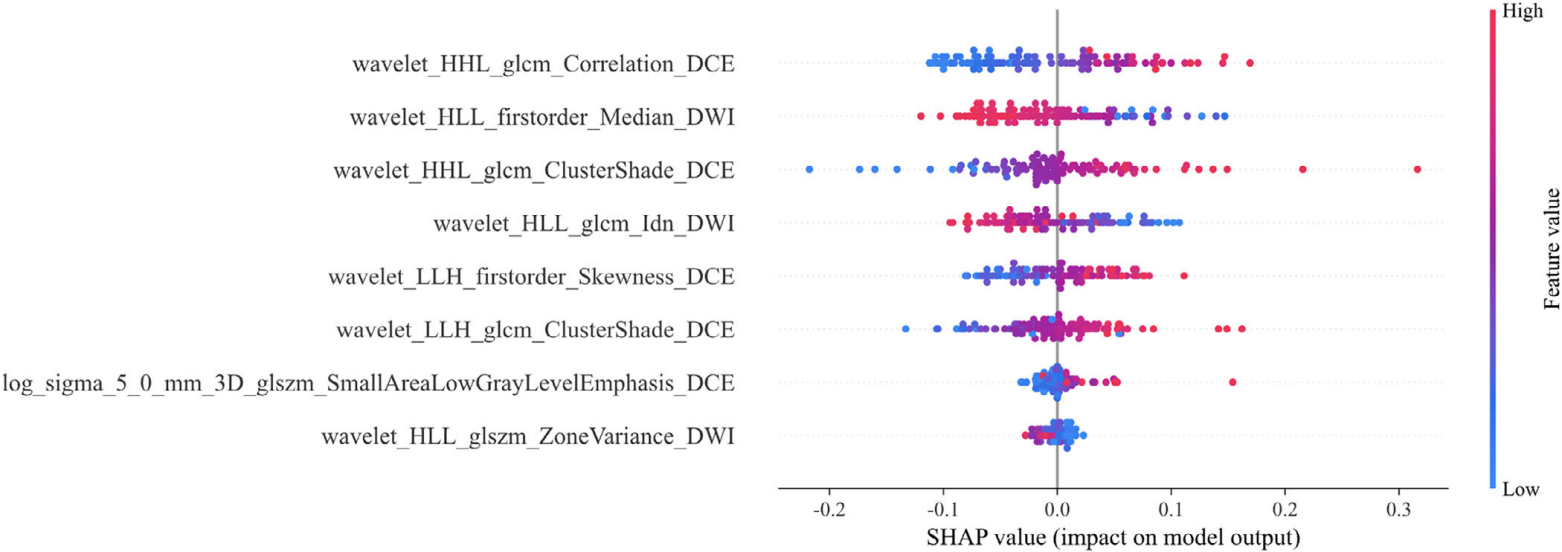
SHAP summary plots of radiomic model. The plot illustrated the feature relevance and attributions to the model's predictive performance.

SHapley Additive exPlanations provided a quantitative interpretation framework for the SVM classifier. SHAP summary plots (Figure 5) visually depicted the impact magnitude and direction of features on model predictions. Features were ranked vertically by global importance. Individual dots represented the SHAP value of each feature for a specific patient, plotted horizontally, and stacked vertically to indicate density distribution. Dot coloration reflected feature values, ranging from low (blue) to high (red). The wavelet_HHL_glcm_Correlation_DCE emerged as the most influential feature for discriminating sensitivity to neoadjuvant chemotherapy in breast cancer. The density spread of its SHAP values indicated variation across the cohort, while the consistent shift towards red showed higher feature values corresponding to increased model output values (higher risk of sensitivity).

**FIGURE 6:**
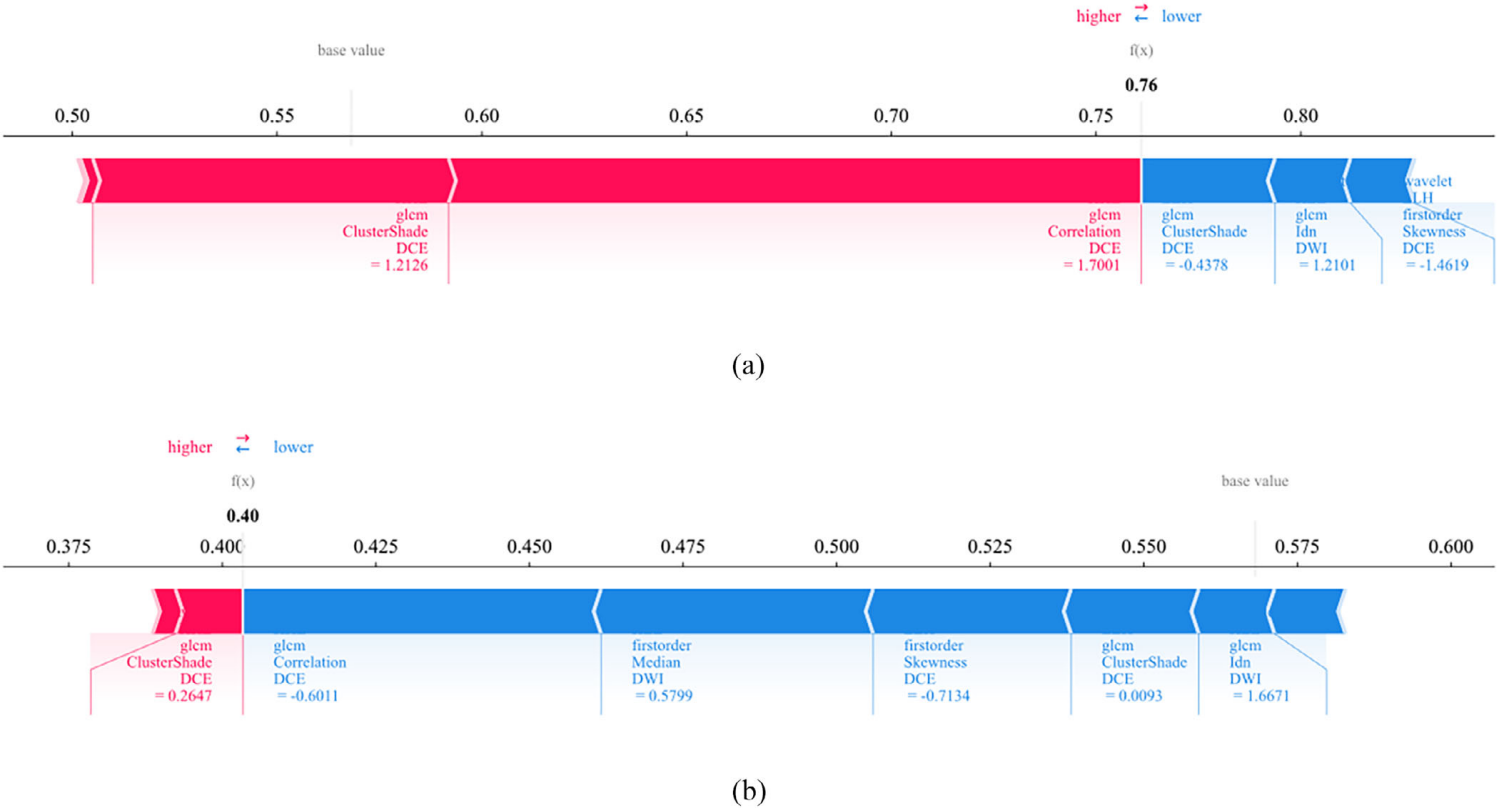
SHAP force plots explained how the radiomic model discriminates the treatment response oftwo patients. The treatment response of patient A was NAC‐sensitive group (A) and the treatment response of patient B was NAC‐insensitive group (B).

Force plots (Figure 6) elucidated individual patient predictions. Each prediction started from the base value (0.568, the mean prediction across all patients). Feature‐specific forces, represented by arrows, either increased (red) or decreased (blue) the prediction value. Arrow length indicated the magnitude of each feature's contribution to the SHAP value. For the patient in Figure 6A, the overall SHAP value (0.76) exceeded the base value, leading to classification as NAC‐sensitive. The high positive SHAP value from wavelet_HHL_glcm_Correlation_DCE (value: 1.7001, red arrow) was a major contributing factor. Conversely, Figure 6B depicts a patient with a SHAP value (0.40) below the base value, resulting in a NAC‐Insensitivity classification. Here, a lower value of wavelet_HHL_glcm_Correlation_DCE (value: −0.6011, blue arrow) significantly decreased the likelihood of a NAC‐insensitive prediction.

## 4 DISCUSSION

Neoadjuvant chemotherapy demonstrates significant clinical value in locally advanced and inoperable breast cancer by facilitating tumor downstaging and improving surgical outcomes.^18^ However, accurately identifying patients likely to benefit from NAC remains challenging. Current evaluation predominantly relies on pathological systems such as the MP grading system, which assesses treatment response through post‐NAC tumor cellularity reduction.^19^ Although MP grading correlates with disease‐free survival,^20^ its retrospective nature limits utility for guiding early clinical intervention. Consequently, imaging evaluations emerge as critical tools for dynamic monitoring and early prediction of NAC efficacy, enabling timely treatment adjustments for non‐responders while optimizing surgical timing for responders.

Radiomics addresses this need by extracting high‐dimensional medical imaging features that reflect tumor heterogeneity at morphological, genetic, and molecular levels.^21^ Previous MRI‐based radiomic studies for NAC response prediction have primarily utilized single sequences (e.g., DCE‐MRI) or limited feature sets.^22^ ^,^ ^23^ While mpMRI radiomics combined with clinical data shows promise in predicting pathological complete response (pCR),^7^ ^,^ ^24^ ^,^ ^25^ research on early identification of NAC‐resistant cases remains scarce. For instance, Liu et al.^7^ reported that an integrated mpMRI radiomic‐clinical model outperformed radiomics‐only approaches in pCR prediction (AUC: 0.860 vs. 0.790), yet NAC sensitivity prediction was not specifically addressed.

This study leverages mpMRI radiomics and clinical variables to develop an interpretable model for early NAC sensitivity prediction. Our findings identify significant associations between NAC sensitivity and radiomic features—particularly wavelet‐transformed texture patterns—alongside progesterone receptor (PR) status and glandular tissue composition. The integrated radiomic‐clinical model achieved robust performance, with AUCs of 0.904 (95% CI: 0.816–0.991) in the internal validation set and 0.928 (95% CI: 0.874–0.982) in the external validation set, surpassing single‐modality approaches. These results align with Tahmassebi et al.’s work demonstrating mpMRI‐based machine learning for pCR prediction,^26^ and with a recent meta‐analysis confirming the superiority of integrated radiomic‐clinical models over unimodal strategies.^27^ We postulate that mpMRI captures complementary biological information—with DCE‐MRI quantifying vascular permeability and DWI reflecting cellular density—enabling more comprehensive response assessment than single‐parameter evaluations.^28^

The resultant nomogram (Figure 4) provides clinicians with an intuitive tool for risk stratification by integrating radiomic signatures (e.g., DWI_DCE radiomics score) with established clinical predictors (ER, PR, HER2 status, glandular features). This visualization tool facilitates: 1) Quantification of individual risk contributions via the Points scale (e.g., high DWI_DCE score contributing > 50 points); 2) Rapid calculation of total risk scores (0–160 scale); and 3) Correlation of scores with predicted insensitivity probability (Risk axis: 0–0.95), thereby translating complex model outputs into actionable clinical decisions.^29^

Despite the robust performance of our SVM‐based radiomic‐clinical model, clinical adoption necessitates transparent decision‐making. We addressed this by implementing SHAP, a game‐theoretic approach that quantifies individual feature contributions to predictions.^30^ SHAP enhances interpretability through two complementary visualizations: 1) Global interpretability: SHAP summary plots (illustrating features like wavelet_HHL_glcm_Correlation_DCE) rank features by overall impact magnitude while color‐coding feature values (low: blue; high: red), revealing how specific feature ranges drive predictions toward insensitivity or sensitivity;^31^ 2) Individualized explanation: SHAP force plots (Figure 6A) visualize how features collectively shift a patient's prediction from baseline probability, with high wavelet_HHL_glcm_Correlation_DCE values (red arrows) strongly favoring sensitivity classification and arrow lengths indicating contribution magnitude.^32^ This methodology enhances clinical utility compared to nomogram scoring systems,^33^ enabling rapid identification of patient‐specific sensitivity drivers.

This study has several limitations. First, the exclusion of tumor differentiation grade and detailed TNM staging may impact model comprehensiveness; future work should incorporate these clinicopathological factors. Furthermore, manual tumor segmentation introduces potential inter‐observer variability; automated deep learning‐based segmentation solutions are currently under investigation.

## 5 CONCLUSION

By integrating mpMRI radiomics with clinicopathological variables and SHAP‐based interpretability frameworks, we established a radiomic‐clinical model that effectively predicted breast cancer patients likely to be sensitive to NAC. This non‐invasive approach provides an early stratification tool for treatment personalization, optimizing therapeutic strategies while sparing patients unnecessary treatment toxicities.

## AUTHOR CONTRIBUTIONS

**Xinyi Zeng**: Writing—review and editing; writing—original draft; visualization; conceptualization; data curation; formal analysis. **Jinxin Chen**: Writing—review and editing; writing—original draft; methodology. **Xianjun Zeng**: Resources; investigation; data curation. **Xiaofei Tang**: Methodology; software; formal analysis. **Jidong Peng**: Writing—review and editing, supervision, project administration, funding acquisition.

## CONFLICT OF INTEREST STATEMENT

The authors declare that they have no known competing financial interests or personal relationships that could have appeared to influence the work reported in this paper.

## ETHICS APPROVAL

The study complied with the Declaration of Helsinki and obtained ethical approval from all participating institutions, with the requirement for patient informed consent waived.

## Supporting information



Supporting Information

## Data Availability

Authors will share data upon request to the corresponding author.
