# Supplementary material for: Integrating multiparametric MRI radiomics and clinical models to assess sensitivity to neoadjuvant chemotherapy in breast cancer: A multicenter study
Source: J Appl Clin Med Phys. 2025 Nov 14;26(11):e70347. doi: 10.1002/acm2.70347 (PMC12618187; doi:10.1002/acm2.70347)
Supplement: Supplementary file 1 — Supporting Information [file ACM2-26-e70347-s001.docx]

Supplementary Figure 1-3. The LASSO algorithm selects the feature path map based on the lesion area and the best retained features. (Figure 1) Mean square error diagram; (Figure 2) Regression coefficient diagram; (Figure 3) Best feature set.

Supplementary Figure 4. Clinical, radiomics and radiomics-clinical calibration curves in the training set (4A), internal validation set (4B), external validation set (4C).

Supplementary Figure 5. Clinical, radiomics and radiomics-clinical decision curves in the training set (5A), internal validation set (5B), external validation set (5C).

Supplementary Figure 6. The result of delong test.
